# Chirality and structure-selective MALDI using mold matrix

**DOI:** 10.1038/s41598-018-31629-6

**Published:** 2018-09-03

**Authors:** Yosuke Fujii, Jiawei Xu, Tatsuya Fujino

**Affiliations:** 1 Department of Chemistry, Graduate School of Science and Engineering, Tokyo Metropolitan University, 1-1 Minami-Osawa, Hachioji, Tokyo 192-0379 Japan; 2 Department of Applied Chemistry, Graduate School of Science and Engineering, Toyo University, 2100 Kujirai, Kawagoe, 350-8585 Japan; 3 Bio-Nano Electronics Research Centre, Toyo University, 2100 Kujirai, Kawagoe, 350-8585 Japan

## Abstract

A matrix that enabled chirality and structure-selective detection in matrix assisted laser desorption ionization mass spectrometry (MALDI MS) has been developed. Molds of L- or D- alanine were made on a thermoreversible polymer (polyvinyl methyl ether) with 2,4,6-trihydroxyacetophenone, and this was used as a matrix. Separate detection of one optical isomer of alanine was realized in MALDI MS. This technique was also applied to the detection of trisaccharides having the same molecular weight but different structures. Separate detection of raffinose and maltotriose in MALDI MS were presented.

## Introduction

The importance of dealing with enantiomers is increasing in many research fields, including medicine, agriculture, and food, because enantiomers exhibit different biological activities. For example, L-glutamic acid has umami flavor but D-glutamic acid does not, and L-ibuprofen is an analgesic but D-ibuprofen is not. One optical isomer exhibits biological activity, whereas another isomer either does not do so or sometimes exhibits minimal activity. It is known that the pharmacological activity of a racemate is often decreased. Therefore, the synthesis, separation, and detection of optical isomers is very important.

Mass spectrometry has shown high potential for the detection of enantiomers. Mass spectrometry used in combination with various separation techniques, including gas chromatography-mass spectrometry (GC-MS)^1^ and liquid chromatography-mass spectrometry (LC-MS)^2,3^, has been employed. Matrix-assisted laser desorption/ionization mass spectrometry (MALDI-MS) is a soft ionization technique for the determination of the molecular weight of an analyte^4–6^. Although MALDI-MS enables rapid and easy detection of an analyte because it can analyze simultaneously a mixture of analyte and adulterants without any time-consuming pretreatments, the separate detection of compounds having the same molecular weight is not possible. The emergence of the ion mobility technique has enabled the detection of compounds having largely different structures^7–9^. The use of a cyclodextrin cavity (CD) has been proposed for the differentiation of isotope-labeled enantiomers^10^ because CD shows different host-guest interactions. However, the separate detection of optical isomers by MALDI-MS is still difficult. In MALDI, the matrix plays a vital role in the ionization as well as the desorption of an analyte. Therefore, not only a conventional matrix, such as an organic acid, a metal or semiconductor nanoparticles, but also a molecular complex having a unique function could be used as a matrix if it had the ability to ionize and desorb an analyte. Herein we present the separate detection of alanine by using a newly developed chirality-sensitive matrix. This technique was applied to the detection of trisaccharides having the same molecular weight but different structures.

## Results and Discussion

**Figure 1:**
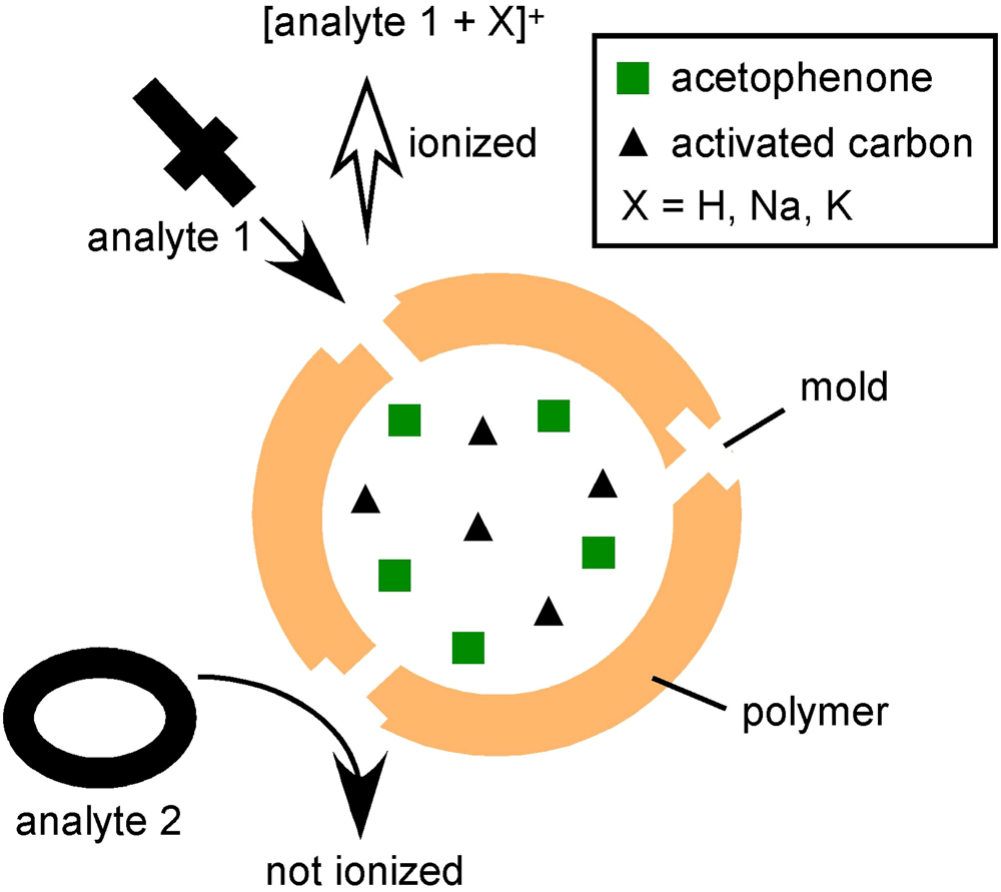
Schematic diagram of chirality-sensitive matrix.

PVME is a polymer that shows thermoreversible phase separation. PVME was dissolved in solution homogeneously at a temperature below the low critical solution temperature (LCST ~ 308 K) where it adopted a coiled structure and its ether oxygen formed hydrogen bonds with water molecules. When the temperature exceeded LCST, the coiled structure became globular and the polymer coils were dehydrated. The polymers in globules aggregated to form fine particles via hydrophobic interactions. Figure 1 shows the schematic diagram of the developed matrix for chirality-sensitive detection. By increasing the temperature of the mixture of PVME, THAP, and L-Ala from room temperature to above LCST, PVME forms a globule while holding L-Ala and THAP in its structure. As L-Ala is water-soluble, L-Ala exists at the outer surface of PVME facing the solution. In contrast, as THAP is hardly soluble in water, THAP exists in the core of PVME globule. Through dialysis, L-Ala is removed from PVME, leaving a mold of its structure. However, even though D-Ala meets this complex, D-Ala would not fit the mold made by L-Ala. Thus, D-Ala would not be accessible to THAP existing in the polymer core and would not be ionized. On the other hand, L-Ala would fit the mold on this complex and thus, would be accessible to THAP and be ionized.

**Figure 2:**
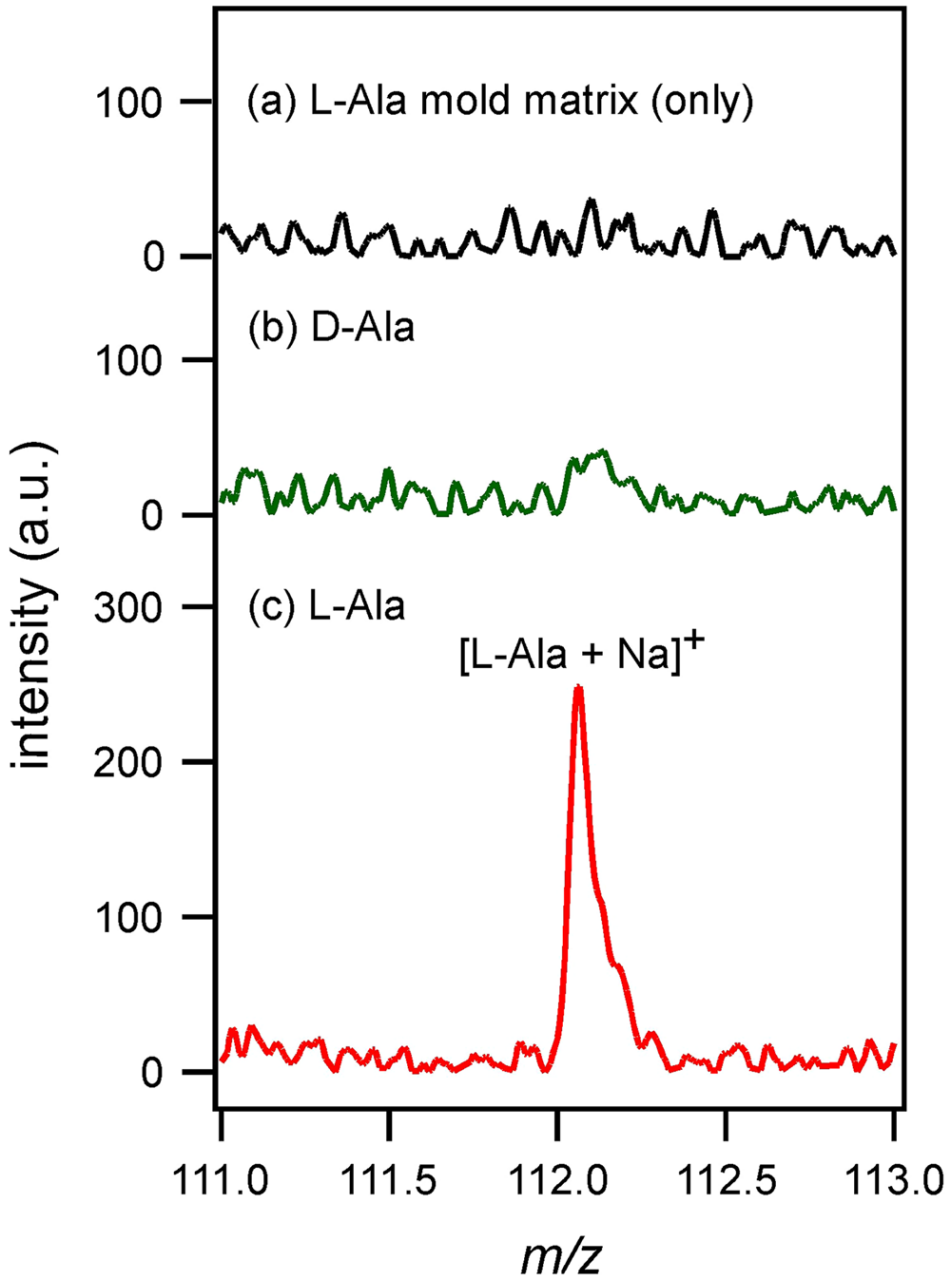
(**a**) Mass spectrum of L-Ala mold matrix only (without any analyte). Mass spectra of (**b**) D-Ala and (**c**) L-Ala measured with L-Ala mold matrix.

Figure 2 shows the results of laser desorption ionization mass spectrometry using the L-Ala mold matrix. When the matrix alone (without any analyte) was subjected to mass spectrometry, no peak due to L-Ala ([L-Ala + Na]^+^) was detected. Therefore, almost all of the L-Ala molecules were removed by dialysis. When D-Ala as the analyte was measured by using the L-Ala mold matrix, the [L-Ala + Na]^+^ peak was not detected, as shown in Fig. 2(b). Although a slight upsurge was found at *m/z* = 112.1, the signal-to-noise ratio (S/N) was below 3 (limit of detection; LOD) and therefore, that upsurge could not be recognized as a peak. On the other hand, when L-Ala was used as the analyte, L-Ala was ionized by THAP and the [L-Ala + Na]^+^ peak was detected. These results showed that the developed matrix enabled chirality-selective detection by laser desorption ionization.

To confirm the practical applicability of the developed matrix, a D-Ala mold matrix was produced next and used to investigate the dependence of matrix chirality-selective detection on analyte concentration. The analyte, which is a mixed solution of D-Ala and L-Ala, was subjected to MALDI MS. The concentration ratio of D-Ala to L-Ala was changed as follows: 1 mg/mL:0 mg/mL, 0.5:0.5, 0.25:0.75, and 0:1, while the total amount of D-Ala and L-Ala was kept constant (1 mg/mL). The results are summarized in Table 1. As mentioned in Methods, the concentrations of THAP and D-Ala used to make the mold matrix were 4 and 0.5 mg/mL, respectively. Therefore, the molds on PVME would be fully filled with 0.5 mg/mL D-Ala as the analyte (5.6 nmol). It was found from Table 1 that the peak intensities at *m/z* = 112.1 were almost the same when the ratios of 1:0 and 0.5:0.5 were used, as the number of molds was limited and fully filled with D-Ala when D-Ala concentration exceeded 0.5 mg/mL as the analyte. However, when the 0.25:0.75 analyte solution was used, the peak intensities at *m/z* = 112.1 were reduced to half as the concentration of D-Ala was halved. Although a slight upsurge was found for the ratio of 0:1, the S/N ratio was below 3 (noise level = 31) and therefore, that upsurge could not be treated as a peak. It was found that the peak intensities at *m/z* = 112.1 were decreased almost linearly according to the concentration change of D-Ala and not L-Ala. Therefore, it was confirmed that the peak at *m/z* = 112.1 was due to [D-Ala + Na]^+^ and developed matrix realized the chirality-selective detection of analyte. From Table 1, the limit of detection (LOD) was calculated to be 1.58 nmol and the average of reproducibility (relative standard deviation; RSD) was 12.17%.

**Table 1:** Peak intensities of [D-Ala + Na]^+^ (*m/z* = 112.1) and standard deviations.

Sample D-Ala:L-Ala	#1	#2	#3	#4	#5	Avg.	SD (a.u.)	RSD (%) intra overall	
1.00: 0.00	314	378	322	396	316	345.2	38.80	11.24	12.17
0.50: 0.50	338	343	360	319	370	346.0	19.87	5.73	
0.25: 0.75	185	162	163	149	190	169.8	17.17	10.11	
0.00: 1.00	47	66	40	67	62	56.4	10.11	21.59	

**Figure 3:**
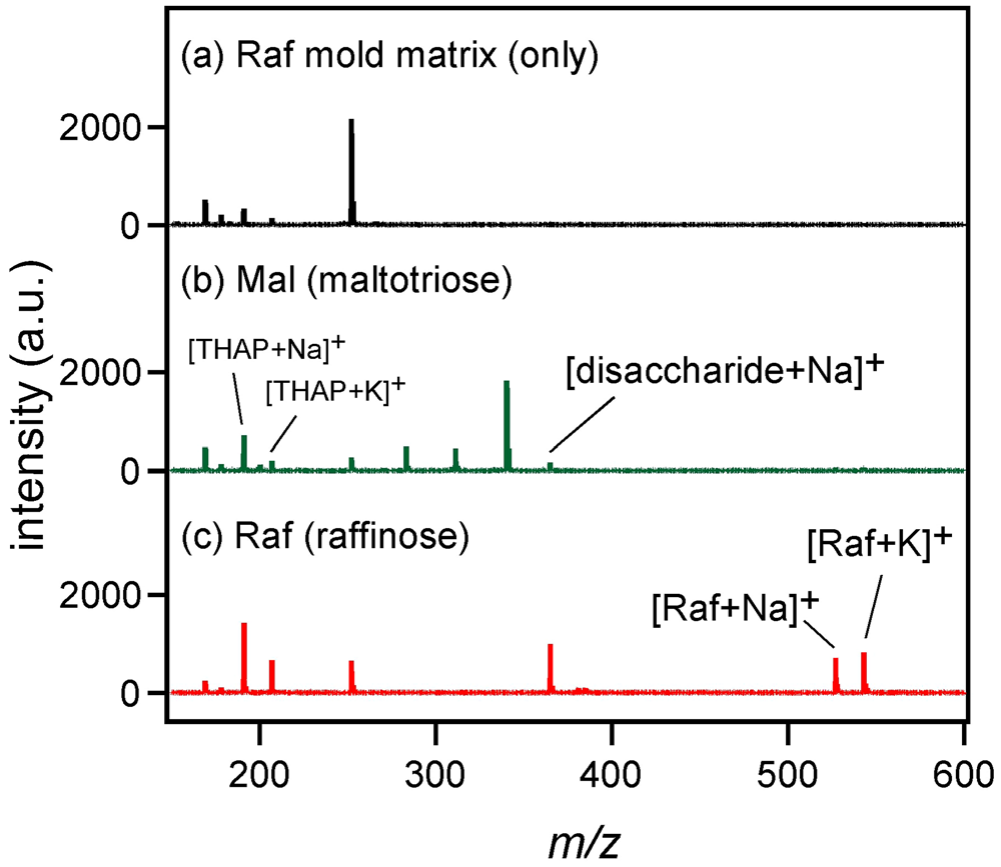
(**a**) Mass spectrum of Raf mold matrix only (without any analyte). Mass spectra of (**b**) Mal and (**c**) Raf measured with Raf mold matrix.

Raffinose (Raf) and maltotriose (Mal) are trisaccharides having the same molecular weight (504.4) but different structures. A matrix was developed with Raf as the mold. In this experiment, activated carbon (AC) was included as well as THAP as the adsorbent of saccharides. Figure 3(a) shows the mass spectrum of Raf mold matrix only (without any analyte). Saccharide–related peaks were not recognized, indicating that Raf used as the mold was completely removed by dialysis. Then, Mal was measured with the Raf mold matrix and the result is shown in Fig. 3(b). Although a disaccharide peak was visible, the Mal peak was not observed. When Raf was used as the analyte, [Raf + Na]^+^ and [Raf + K]^+^ peaks were observed at *m/z* = 527.4 and 543.4, indicating that molecular structure sensitive laser desorption ionization was achieved by using the mold matrix.

## Conclusions

A matrix that enabled chirality- and structure-selective detection in MALDI MS has been developed. By making a mold of L-Ala or D-Ala in the thermoreversible polymer (PVME), separate detection of one optical isomer of alanine was realized. This technique was applied to detection of trisaccharides having the same molecular weight but different structures. Separate detection of raffinose and maltotriose were presented.

## Methods

Activated carbon (AC), 2,4,6-trihydroxyacetophenone (THAP), raffinose (Raf), and maltotriose (Mal) were obtained from Wako. Polyvinyl methyl ether (PVME) and D- and L-alanine (D-Ala and L-Ala) were obtained from Sigma. They were used without further purification. For the chirality-selective MALDI, THAP and L-Ala (1:0.125 wt. ratio) were mixed in a mortar and pestle. The mixture (80 mg) was dissolved in PVME solution (10 wt%; ethanol:water = 4:1) (20 mL) such that the concentration of THAP was 4 mg/mL. The obtained solution (5 mL) was transferred into a dialysis tube and dialyzed for ten minutes in hot water (323 K) to release L-Ala. The dialysis was repeated three times. For the structure-selective MALDI, AC, THAP, and Raf (1:1:1 wt. ratio) were mixed in a mortar and pestle. The mixture (120 mg) was dissolved in PVME solution (20 wt%; ethanol:water = 4:1) (20 mL) and the obtained solution was subjected to dialysis in the same manner as Ala. One microliter of analyte solution (1 mg/mL) was pipetted onto a sample plate whose temperature was kept at 323 K, and then one microliter of the solution after dialysis (matrix solution) was pipetted. After the evaporation of solvent, the sample plate was placed inside a commercial MALDI/TOF (Bruker), and mass spectrometry was carried out.
